# Does Mode of Surgical Intervention Based on Oncotype DX Score Influence Disease Recurrence in Early Breast Cancer?

**DOI:** 10.1055/s-0040-1712537

**Published:** 2020-06-19

**Authors:** T. M. Aherne, M. R. Boland, D. Catargiu, K. Bashar, T. P. McVeigh, C. Brodie, K. J. Sweeney

**Affiliations:** 1 Department of Breast Surgery, University Hospital Galway, Galway, Ireland; 2 Department of Pathology, University Hospital Galway, Galway, Ireland; 3 Royal College of Surgeons in Ireland, Stephens Green, Dublin, Ireland; 4 BreastCheck, Western Unit, Galway, Ireland

**Keywords:** Oncotype DX, early breast cancer, recurrence, surgery

## Abstract

**Introduction** Routine utilization of multigene assays to inform operative decision-making in early breast cancer (EBC) treatment is yet to be established. In this pilot study, we sought to establish the potential benefits of surgical intervention in EBC based on recurrence risk quantification using the Oncotype DX (ODX) assay.

**Materials and Methods** Consecutive ODX tests performed over a 9-year period from October 2007 to May 2016 were evaluated. Oncotype scores were classified into high (≥31), medium (18–30), or low-risk (0–17) groups. The primary outcome was breast cancer recurrence. Subgroup analysis offered assessment of the recurrence effect of mode of surgical intervention for patient groups as defined by the oncotype score.

**Results** In total 361 patients underwent ODX testing. The mean age and follow-up were 55.25 (± 10.58) years and 38.59 (± 29.1) months, respectively. The majority of patients underwent wide local excision (86.7%) with 8.9 and 4.4% patients having a mastectomy or wide local excision with completion mastectomy, respectively. Fifty-one percent of patients fell into the low risk ODX category with a further 40.2 and 8.5% deemed to be of intermediate and high risk. Five patients (1.38%) had disease recurrence. Comparative analysis of operative groups in each oncotype group revealed no difference in recurrence scores in the low- ( *p* = 0.84) and high-risk groups ( *p* = 0.92) with a statistically significant difference identified in the intermediate risk group ( *p* = 0.002).

**Conclusion** To date we have been unable to definitively identify a role for ODX in guiding surgical approach in EBC. There is, however, a need for larger studies to examine this hypothesis.

Multigene tumor assays (MGAs) have offered a greater insight into patient prognosis in early, node negative, hormone receptor positive breast cancers. This further understanding of tumor genetics has allowed oncologists and breast surgeons alike to tailor adjuvant therapy more specifically to individual patients.

Despite the proven benefit of adjuvant chemotherapy (AC), 1 2 3 a large proportion of early breast cancer patients is exposed to its negative effects unnecessarily. 4 In recent years, MGAs have guided the use of AC in this cohort resulting in a significant reduction in the AC-related morbidity. 5 In addition, it provides objective, validated data 6 promoting a standardized approach to the management of these cancers across centers globally with its utilization supported by the American Society of Clinical Oncology. 7

Despite the evolution of systemic directed therapy, locoregional control through surgical intervention and adjuvant radiotherapy remain the mainstay of treatment with proven prognostic benefits. 8

The specific MGA, Oncotype DX (ODX) 21-gene recurrence score, offers a widely accepted tool to guide prognosis and predict chemotherapy efficacy in this group. Furthermore, the ODX breast cancer assay for ductal carcinoma in situ 9 (DCIS) has emerged as a useful adjunct for predicting patients who stand to benefit from post-breast conservation surgery radiotherapy. 10 11

However, the routine utilization of MGAs to inform operative decision-making is yet to be established with a paucity of published data. In this pilot study, we sought to identify the relationship of the ODX score and mode of surgical intervention (breast conserving and mastectomy) with a view to establishing the potential benefits of intervention based on recurrence risk quantification. We hypothesize that ODX may predict the prognostic advantages of various surgical interventions and hence inform initial operative decision-making.

## Materials and Methods

### Study Design and Participants

In this retrospective cohort study, all patients with stage T1 or T2, node negative, estrogen receptor positive breast cancer undergoing ODX testing were included for analysis. Consecutive ODX tests performed over a 9-year period from October 2007 to May 2016 were evaluated. Study design was guided by the STROBE guidelines for reporting observational trials 12 with full ethical approval granted by the Galway Clinical Research and Ethics Committee.

Pathological assessment of each tumor specimen was performed in the Department of Pathology at University Hospital Galway. All cases were discussed in a multidisciplinary (MDT) setting in the presence of at least one breast surgeon, radiologist, and pathologist. Suitable cases, as described, were referred for ODX scoring with testing performed by Genomic Health Laboratory, Redwood City, California using an established protocol. 2 Oncotype scores were classified as high (≥31), medium (18–30), or low risk (0–17) as defined by Paik et al. 6 Adjuvant management was then determined following further MDT and patient consultation.

### Outcomes

The primary outcome was breast cancer recurrence. Recurrence was confirmed locally or distantly with either histological or radiological confirmation. Further subgroup analysis assessed the recurrence effect of mode of surgical intervention for patient groups as defined by the oncotype score. Secondary end points included tumor characterization, adjuvant therapeutic stratification, and reintervention rates.

### Data

Patient demographic, follow-up, recurrence, and adjuvant therapy data were extracted from the regional Oncology Management System (Citrix Systems Inc. Santa Clara, CA). Tumor detail was obtained from hospital pathology reports. Missing data were identified from review of patient hospital notes. For comparative purposes, patients were cohorted into low-, medium-, and high-risk oncotype score groups. Normally distributed data were reported as mean (standard deviation), while outcome data were reported nominally (percentage) and compared using the chi-squared test. All data were tabulated using Microsoft Excel (Microsoft Corporation, Redmond, WA) and Statistical Package for the Social Sciences (SPSS) software (SPSS Inc., Chicago, IL) was used for data analysis.

## Results

### Demographic Data

In total 361 patients underwent oncotype testing over the study period with outcome data available for 353. The mean age was 55.25 years (±10.58). Mean follow-up was 38.59 months (±29.1). All tumors were both hormone receptor positive and HER2, node negative. Mean tumor size was 21.64 mm (±9.05). Further tumor pathological characteristics are summarized in Table 1 .

**Table 1:** Tumor pathological characteristics

Tumor subtype ( *n* [%])	Lobular	57 (16)
	Ductal	289 (80)
	Mixed	2 (0.5)
	Tubular	3 (0.8)
	Colloid	6 (1.6)
	Other	4 (1.1)
Grade ( *n* * [%])	1	40 (11)
	2	231 (64)
	3	90 (25)

### Surgical and Adjuvant Intervention

The majority of patients underwent a breast conserving wide local excision (WLE) ( *n* = 313, [86.7%]) of the tumor. A further 32 (8.9%) and 16 (4.4%) patients had either a mastectomy or WLE with completion mastectomy, respectively. Of note, 52 (14.4%) patients required reintervention for either a re-excision of margins ( *n* = 36) or completion mastectomy ( *n* = 16).

With regard to overall adjuvant therapy, 148 patients (40.9%) received chemotherapy with 333 (92.2%) undergoing radiotherapy. Three-hundred and fifteen patients (87.2%) undertook regular systemic hormonal therapy. Adjuvant treatment according to oncotype risk group is outlined further in Table 2 .

**Table 2:** Patient, tumor, and treatment characteristics as defined by oncotype risk score

Oncotype risk group	Low	Medium	High
Patient number	181	142	30
Age	55.1	55.3	55.9
Tumor size (mm)	22.2	20.6	23.25
Grade			
1	29	10	0
2	122	95	8
3	30	37	22
Follow-up (months)	37.1	41.4	37.5
Mode of surgery ( *n* [%])			
Wide local excision	154 (85)	125 (88)	28 (93.3)
Mastectomy	19 (10.5)	10 (7)	1 (3.3)
Wide local excision with completion mastectomy	8 (4.5)	7 (5)	1 (3.3)
Surgical reintervention (%)	15.4	12.6	16.6
Adjuvant therapy ( *n* [%])			
Chemotherapy	21 (11.6)	99 (69.7)	28 (93.3)
Radiotherapy	159 (87.8)	125 (88.65)	30 (100)
Hormonal therapy	160 (88.3)	128 (90.1)	27 (93.1)

Note: All figures are means unless otherwise stated. *n* , number, mm, millimeters.

### Oncotype Score

Three-hundred and fifty-three patients had a documented oncotype score over the study period. When stratified according to recurrence risk, the majority of patients had a “low risk” score ( *n* = 181, [51.3%]). A further 40.2% ( *n* = 142) patients were deemed to be of intermediate risk with a minority [ *n* = 30, (8.5%)] having a high-risk score. Risk groups as defined by oncotype score are further described in Table 2 .

### Disease Recurrence

In total five patients (1.38%) had disease recurrence at a mean of 43.4 months (range: 19–82 months). One patient experienced local recurrence with four further patients developing distant recurrence. Three further patients were diagnosed with DCIS of the contralateral breast during follow-up surveillance.

Subgroup analysis assessing the recurrence effect of mode of surgical intervention for patient groups as defined by the oncotype score is summarized in Table 3 . Disease recurrence was 6.66% (2/30) in the high-risk oncotype group with 0.7 (1/142) and 1.1% (2/181) rates of recurrence in the medium- and low-risk oncotype groups, respectively. Two patients undergoing WLE in both the high- and low-risk groups experienced recurrence with one further recurrence identified in the intermediate group following mastectomy. Comparative analysis of operative groups in each oncotype cohort revealed no difference in disease recurrence in the low- ( *p* = 0.84) and high-risk groups ( *p* = 0.92) with a statistically higher rate of recurrence identified in the intermediate-risk mastectomy group ( *p* = 0.002).

**Table 3:** A comparison of disease recurrence among operative groups as defined by oncotype score

Oncotype score	Mode of surgery	Follow-up	Recurrence ( *n* /total)	Subgroup analysis ( *p* -Value)
<18	WLE	37.4	2/154	
	Mastectomy	35.2	0/9	
	WLE with completion mastectomy	36.9	0/8	0.84
18–31	WLE	41.5	0/125	
	Mastectomy	36.7	1/10	
	WLE with completion mastectomy	47.1	0/7	0.002
>31	WLE	37.0	2/28	
	Mastectomy	59.0	0/1	
	WLE with completion mastectomy	30.0	0/1	0.92

Abbreviations: *n* , number; WLE, wide local excision.

Note: *p* -Value represents the comparative analysis of the recurrence effect of mode of surgical intervention for patient groups as defined the oncotype score.

## Discussion

This study examines the influence of various surgical interventions, based on oncotype risk group, on breast cancer recurrence. Additionally, it further defines the early breast cancer cohort in the genetically diverse West of Ireland. Over the past decade, ODX has emerged as a reliable, externally validated 6 13 tool in the management of this patient group allowing effective individualization of therapy. This study aimed to clarify the potential role for ODX in guiding surgical approach.

The majority of early breast cancers consisted of Grade 2 invasive ductal carcinomas with a low overall recurrence rate (1.38%). Adjuvant therapy in the form of radiotherapy and systemic hormone therapy remained consistent across all risk groups. However, the use of AC varied from 11.6% in the low-risk group to 93.3% in the high-risk oncotype group. These figures are in keeping with previous evidence from the region. 14 A majority of patients in all groups underwent WLE with minorities undergoing mastectomy or WLE with completion. As such, this study remains inadequately powered to definitively identify a role for ODX in determining the surgical approach to these early breast cancers.

The study itself is subject to certain limitations. It is single-centered and retrospective and thus subject to the inherent limitations associated with such studies. While a relatively large sample of 353 patients underwent oncotype testing, recurrence was infrequent and as such standard statistical methods including cox hazard ratios were not incorporated. Furthermore, a majority of recurrences occur after 5 years of endocrine treatment. 15 16 In keeping with international figures, recurrence rates in the study cohort were less than 3% exposing the study to potentially being underpowered. It is, however, unique in its analysis of ODX testing to evaluate the merits of each surgical mode and the authors would expect larger multicenter studies to add to this knowledge as the role of MGAs develops.

While the management of early breast cancer continues to evolve, local disease control in the form of surgery and radiotherapy remains the cornerstone of therapy. Meta-analysis data has suggested that mode of locoregional control directly influences disease recurrence based on luminal classification. 17 18 As such tumor subtypes with increased metastatic potential should invariably be approached with more aggressive local intervention. ODX offers an effective tool in further characterizing node negative, hormone receptor positive breast cancers. It has been widely effective in tailoring both chemotherapy and radiotherapy prescription thus limiting disease recurrence in high-risk patients and the debilitating side effects of adjuvant therapy in the lower-risk cohort. 5 10 11 The use of MGAs has significant potential to guide mode of surgical intervention; however, to date its merits are yet to be established in the absence of larger multicenter trials.

## Conclusion

While MGA, an effective, accessible tool, has offered treating physicians a greater understanding of breast cancer genetics, it may well remain underutilized. In this pilot study, we sought to identify the relationship of the ODX score and mode of surgical intervention with a view to establishing the potential benefits of intervention based on recurrence risk. To date we have been unable to definitively identify a role for ODX in guiding surgical approach. There is, however, a need for larger multicenter studies examining this hypothesis.

## References

[1] Fisher B Costantino J Redmond C A randomized clinical trial evaluating tamoxifen in the treatment of patients with node-negative breast cancer who have estrogen-receptor-positive tumors N Engl J Med 1989 320 08 479 484 2644532 10.1056/NEJM198902233200802

[2] Fisher B Jeong J H Bryant J Treatment of lymph-node-negative, oestrogen-receptor-positive breast cancer: long-term findings from National Surgical Adjuvant Breast and Bowel Project randomised clinical trials Lancet 2004 364(9437):858 868 15351193 10.1016/S0140-6736(04)16981-X

[3] Fisher B Dignam J Wolmark N Tamoxifen and chemotherapy for lymph node-negative, estrogen receptor-positive breast cancer J Natl Cancer Inst 1997 89 22 1673 1682 9390536 10.1093/jnci/89.22.1673

[4] Early Breast Cancer Trialists' Collaborative Group (EBCTCG).Effects of chemotherapy and hormonal therapy for early breast cancer on recurrence and 15-year survival: an overview of the randomised trials Lancet 2005 365(9472):1687 1717 15894097 10.1016/S0140-6736(05)66544-0

[5] Paik S Tang G Shak S Gene expression and benefit of chemotherapy in women with node-negative, estrogen receptor-positive breast cancer J Clin Oncol 2006 24 23 3726 3734 16720680 10.1200/JCO.2005.04.7985

[6] Paik S Shak S Tang G A multigene assay to predict recurrence of tamoxifen-treated, node-negative breast cancer N Engl J Med 2004 351 27 2817 2826 15591335 10.1056/NEJMoa041588

[7] Harris L N Ismaila N McShane L M Use of biomarkers to guide decisions on adjuvant systemic therapy for women with early-stage invasive breast cancer: American Society of Clinical Oncology Clinical Practice Guideline J Clin Oncol 2016 34 10 1134 1150 26858339 10.1200/JCO.2015.65.2289 PMC4933134

[8] Darby S McGale P Correa C Effect of radiotherapy after breast-conserving surgery on 10-year recurrence and 15-year breast cancer death: meta-analysis of individual patient data for 10,801 women in 17 randomised trials Lancet 2011 378(9804):1707 1716 22019144 10.1016/S0140-6736(11)61629-2 PMC3254252

[9] Solin L J Gray R Baehner F L A multigene expression assay to predict local recurrence risk for ductal carcinoma in situ of the breast J Natl Cancer Inst 2013 105 10 701 710 23641039 10.1093/jnci/djt067 PMC3653823

[10] Alvarado M Carter D L Guenther J M The impact of genomic testing on the recommendation for radiation therapy in patients with ductal carcinoma in situ: a prospective clinical utility assessment of the 12-gene DCIS score™ result J Surg Oncol 2015 111 08 935 940 26031501 10.1002/jso.23933

[11] Rakovitch E Nofech-Mozes S Hanna W A population-based validation study of the DCIS Score predicting recurrence risk in individuals treated by breast-conserving surgery alone Breast Cancer Res Treat 2015 152 02 389 398 26119102 10.1007/s10549-015-3464-6 PMC4491104

[12] von Elm E Altman D G Egger M Pocock S J Gøtzsche P C Vandenbroucke J P; STROBE Initiative.The Strengthening the Reporting of Observational Studies in Epidemiology (STROBE) statement: guidelines for reporting observational studies Lancet 2007 370(9596):1453 1457 18064739 10.1016/S0140-6736(07)61602-X

[13] Habel L A Shak S Jacobs M K A population-based study of tumor gene expression and risk of breast cancer death among lymph node-negative patients Breast Cancer Res 2006 8 03 R25 16737553 10.1186/bcr1412 PMC1557737

[14] McVeigh T P Hughes L M Miller N The impact of Oncotype DX testing on breast cancer management and chemotherapy prescribing patterns in a tertiary referral centre Eur J Cancer 2014 50 16 2763 2770 25240289 10.1016/j.ejca.2014.08.002 PMC4204201

[15] Regan M M Neven P Giobbie-Hurder A Assessment of letrozole and tamoxifen alone and in sequence for postmenopausal women with steroid hormone receptor-positive breast cancer: the BIG 1-98 randomised clinical trial at 8·1 years median follow-up Lancet Oncol 2011 12 12 1101 1108 22018631 10.1016/S1470-2045(11)70270-4 PMC3235950

[16] Cuzick J Sestak I Baum M Effect of anastrozole and tamoxifen as adjuvant treatment for early-stage breast cancer: 10-year analysis of the ATAC trial Lancet Oncol 2010 11 12 1135 1141 21087898 10.1016/S1470-2045(10)70257-6

[17] Lowery A J Kell M R Glynn R W Kerin M J Sweeney K J Locoregional recurrence after breast cancer surgery: a systematic review by receptor phenotype Breast Cancer Res Treat 2012 133 03 831 841 22147079 10.1007/s10549-011-1891-6

[18] Wang J Xie X Wang X Locoregional and distant recurrences after breast conserving therapy in patients with triple-negative breast cancer: a meta-analysis Surg Oncol 2013 22 04 247 255 24144808 10.1016/j.suronc.2013.10.001

